# Exploring quality of life post sudden onset hearing loss: A convergent parallel approach

**DOI:** 10.4102/sajcd.v71i1.990

**Published:** 2024-02-16

**Authors:** Liepollo Ntlhakana, Sabeehah Hamid

**Affiliations:** 1 Department of Audiology, Faculty of Humanities, University of the Witwatersrand, Johannesburg, South Africa

**Keywords:** sudden onset hearing loss, quality of life, counselling, convergent parallel, family-centred care, adults

## Abstract

**Background:**

Sudden onset hearing loss (SOHL) is rare and presents differently to individuals; hence, it is complex to diagnose. The impact on the quality of life (QoL) varies for individuals and their support structure. However, the exploration of research designs is warranted.

**Objectives:**

This study explored the lived experiences of adults post-SOHL diagnosis and the impact on the QoL. Facilitators of emotional and social aspects of counselling provided by audiologists post-SOHL diagnosis were established.

**Method:**

This was a convergent parallel research study. Data were collected from the two primary participants and three secondary participants, face-to-face and telephonically. The Hearing Handicap Inventory for Adults (HHIA) screening tool and the semi-structured interviews were used for data collection. The data sets were analysed independently, *viz*. descriptive analysis and thematic analysis, to confirm the impact on the QoL post-SOHL diagnosis.

**Results:**

The HHIA scores obtained were 84% and 50% for P1 and P2, respectively. Key themes that emerged from the interviews revealed that communication difficulties mostly impacted the QoL, which in turn influenced their mental and social well-being. Aural rehabilitation was perceived as ineffective support, thus the inability to reduce the impact on the QoL post-SOHL diagnosis.

**Conclusion:**

The integrated findings indicated the impact on the QoL post-SOHL diagnosis. Convergent parallel methods should be considered by researchers to understand rare auditory pathologies and their impact on the QoL.

**Contribution:**

Person-centred care (PCC) and family-centred care (FCC) are facilitators of counselling that audiologists can employ as QoL management strategies post-SOHL diagnosis.

## Introduction

Sudden onset of hearing loss (SOHL) negatively impacts the most valuable physical sense that is essential for the individual’s connection to the world and to other humans through communication (Gondim et al., 2012). Any subsequent deficit or irregularity in the hearing system which leads to hearing impairment (HI), at any stage of life, may also reduce the quality of an individual’s communication processes, which in turn impacts one’s quality of life (QoL) (Dalton et al., 2003; Gondim et al., 2012; Lin & Albert, 2014). Quality of life is defined as the individual’s perceived physical and mental well-being that may be influenced by other factors (Bakas et al., 2012), for example, HI. Hearing impairment has such a high prevalence globally that in some cases, it has been termed an ‘invisible condition’ and even a ‘silent pandemic’, and it is the most common sensory-neural deficit associated with age-related hearing loss (WHO, 2021). South Africa is said to have a well-established deaf community, with approximately more than 4 million deaf and hard-of-hearing individuals (National Month of Deaf People, 2020).

Sudden onset hearing loss (SOHL) refers to the rapid loss of hearing (a loss of ≥30 dB) all at once, for at least three consecutive frequencies, overnight or over a few days, and it is informally termed ‘sudden deafness’ (Chau et al., 2010; Schreiber et al., 2010). Various aetiologies of SOHL result in different but rare types of hearing loss ranging from simple and reversible to profound and permanent (Alexander & Harris, 2013; Elmoursy et al., 2023; Foden et al., 2013). The majority of SOHL cases are classified as idiopathic, meaning that there is no definite or identifiable cause of the hearing loss (Elmoursy et al., 2023; Kuhn et al., 2011). Some known possible causes of severe SOHL include head injury or trauma, ototoxicity, neural conditions or viral infections such as bacterial meningitis or mumps (Elmoursy et al., 2023; Frosolini et al., 2022; Kuhn et al., 2011).

There was limited research available on SOHL globally. In the USA, sudden sensorineural hearing loss (SSNHL) affects 27 per 100 000 people, and annually, more than 66 000 cases of hearing loss were reported (Frosolini et al., 2022). Seemingly, fewer studies were available in contexts in low-to-middle-income countries (LMICs) (Elmoursy et al., 2023). Despite the dearth of literature, SSNHL may be a factor associated with the increased incidence and prevalence of HI in LAMICs. This may lead to a subsequent lack of adequate reporting of the lived experiences of adults who have been diagnosed with SOHL and the associated QoL post-diagnosis.

The most effective method to counteract the negative effects of any hearing loss is counselling (Pearson et al., 2019). Counselling from an audiologist’s perspective is mainly *via* aural rehabilitation that involves counselling about hearing loss and providing coping strategies to individuals diagnosed with hearing loss and those within their microsystem (Pearson et al., 2019). Information and emotional counselling used to collect client-specific data and analysis of that data by the International Classification of Functioning, Disability and Health (ICF model) were essentially used to determine the contextual factors to be considered in aural rehabilitation (Scarinci et al., 2009; WHO, 2013). Thus, patient-reported outcome measures (PROMs) such as the Hearing Handicap Inventory for the Elderly (HHIE) (Ventry & Weinstein, 1983) and the Hearing Handicap Inventory for the Adults (HHIA) (Newman et al., 1990) conducted post-HI diagnosis and during counselling provided a patient-and-family-centred prognostic information to the individual diagnosed and (ASHA, 2020). Therefore, this study aims to provide insight into the lived experiences and impact on the QoL of individuals with SOHL whilst establishing facilitators of emotional and social aspects of counselling post-diagnosis.

However, context-specific and evidence-based research is unknown, yet crucial in this case, specific to the South African context, as the experiences of an average South African adult diagnosed with HI were compounded by a triple burden of factors, for example, unemployment, limited access to basic hearing healthcare and food security (Joubert & Botha, 2019). Thus, it is important to investigate the impact of an SOHL on an average South African individual as it may manifest differently and perhaps more severely in comparison to other cases reported in Western and high-income countries. It is this very absence of contextually relevant literature and understanding of SOHL that motivates the aim of this study.

## Methods

### Aim

This study aimed to explore the quality-of-life post-onset of SOHL whilst investigating effects on social, emotional and situational subscales to gain a multi-faceted understanding of this complex diagnosis.

### Objectives

The study objectives were: (1) to explore the lived experiences of adults diagnosed with SOHL and (2) to investigate the impact of SOHL on the QoL of individuals diagnosed with the hearing impairment and to establish facilitators of emotional and social aspects of counselling provided by audiologists post-SOHL diagnosis.

### Study design

This was a convergent parallel research study (Creswell & Plano Clark, 2011; Demir & Pismek, 2018). The researchers adopted the use of a convergent parallel design whereby a quantitative survey and qualitative interviews were concurrently conducted to gain a more in-depth insight and multifaceted understanding of the experiences of a small number of individuals diagnosed with SOHL, which is considered rare, and the impact on their QoL (Creswell & Plano Clark, 2011; Demir & Pismek, 2018).

### Participants

Purposive non-probability sampling was used to identify participants who met the study inclusion criteria (Table 1). Participants included in the study were recruited from the Wits University Speech and Hearing Clinic (USHC). There were only two participants who were identified as individuals diagnosed with SOHL in the audiology files accessed from the study site. These participants were labelled and anonymised as primary participants 1 and 2 (P1 and P2). In addition, there were three secondary participants (S1, S2 and S3), S1 (P1’s nephew), S2 (P1’s colleague) and S3 (P2’s wife); these individuals were nominated by the primary participants as their significant others in their microsystem (Granberg et al., 2014). Secondary participants S1 and S2 were related to P1, and S3 was related to P2. Table 1 illustrates the inclusion criteria used for both the primary and secondary participants.

**TABLE 1:** Inclusion criteria for the study.

Primary participant criteria	Secondary participant criteria
Diagnosed with hearing loss of 30 dB (or greater) on a minimum of 3 consecutive frequencies that occurred within 72 h.	Should be nominated by the primary participants and be considered as part of their microsystem which is inclusive of their immediate surroundings and immediate relationships/friendships.
Should be 18 or older to be classified as an adult in South Africa.	-
Should reside in Johannesburg, South Africa. Should have access to a form of technology (smartphone or laptop/computer) that allows for virtual interviews to be conducted on an electronic platform in the form of a video recorded call OR be available to conduct in-person interviews that were in accordance with the current COVID protocols.	Participants who were part of the primary participant’s microsystem prior to SOHL diagnosis and who were still in close relation to the primary participant post-SOHL diagnosis.

SOHL, Sudden onset hearing loss; COVID, coronavirus disease.

### Data collection

Our study site is the audiology clinic that offers hearing healthcare services and is also intended for students’ clinical training. Prior to conducting the study, official permission was granted by the site’s Head of Department to have access to the audiology clinic patients’ records from January 2014 to June 2022 to identify patients diagnosed with SOHL. The selected period was based on the availability of records at the study site. The researchers identified five potential primary participants from the audiology clinic patients’ records, and these potential participants were contacted, by the audiology clinic administrator, either telephonically or *via* email to briefly inform them of the research study and what it entails. Individuals who had agreed to participate in the research gave consent to participate in the study.

### Data collection process

The data collection process included a convergent parallel method. The researchers concurrently conducted a quantitative survey and qualitative interviews as part of the data collection process whereby: (1) Primary participants 1 and 2 completed the Hearing Handicap Inventory Screening Questionnaire for Adults (HHIA) (Newman et al., 1990), where quantitative data were collected. (2) Semi-structured interviews were conducted face-to-face with the P1 and P2, and telephonically with S1, S2 and S3, where qualitative data were collected. The HHIA is a 25-item hearing handicap survey questionnaire adapted from Ventry and Weinstein (1983) by Newman et al. (1990) and was a modified version of the HHIE (Ventry & Weinstein, 1983). For the purpose of the study, a 10-item hearing handicap screening tool was used (Newman et al., 1990). It was deemed as a suitable tool to determine the self-perceived hearing handicap of individual study participants (P1 and P2) who were actively involved in their workspaces. Quantitative scores for individual participants were calculated from the HHIA screening tools.

Next, the semi-structured self-designed interviews (Appendix 1) were designed using various literature sources that focused on the impact of SOHL on the QoL. The qualitative interviews were administered virtually *via* video-recorded calls on MS Teams and Zoom to collect data from the recruited participants (P1, P2, S1, S2 and S3) to determine the impact on the QoL for individuals diagnosed with SOHL. Previous researchers reported that hearing handicap screening tools such as the HHIA may not be used alone as they tend to provide inconsistent patient-to-patient results and their validity was questioned; thus, the designed interview schedule was guided by the HHIA screening tool. These data collection methods in our study were weighed equally but analysed as two independent components to inform an integrated interpretation of the study findings (Creswell & Plano Clark, 2011; Demir & Pismek, 2018). In addition, to improve the scientific rigour of the study, member checking was performed following the interviews.

### Data analysis

For data analysis, the HHIA quantitative scoring was used to show handicap levels obtained, and thereafter, a deductive thematic analysis that followed Braun and Clarke’s (2012) six steps was used to analyse data from interviews. The convergent mixed methods design was used in this study to determine whether HHIA scores and data collected from the semi-structured interview were in agreement (Crowe et al., 2011). Thus, quantitative scores obtained from the HHIA screening tool were analysed separately from the qualitative data obtained from the thematic analysis; thereafter, the HHIA scores were obtained, and the deducted themes were checked for confirmability regarding the impact of SOHL on the QoL (Braun & Clarke, 2012; Korstjens & Moser, 2017).

### Ethical considerations

Prior to the study being conducted, it was obligatory that the researcher attained ethical clearance. Ethical clearance was obtained from the University of the Witwatersrand Human Research Ethics Committee (HREC) (Medical), Protocol Number: M220348. All participants consented to take part in the research study; their identities were protected and raw data obtained from interviews were confidentially kept and protected. Participants’ responses were autonomous and respected by researchers.

## Results

This study explored the impact on the QoL and lived experiences post-SOHL, on participants in Johannesburg, South Africa. The findings were obtained through the HHIA screening tool and the semi-structured interviews, respectively. Table 2 illustrates the demographic information and audiological characteristics of the primary participants.

**TABLE 2:** Demographic information, clinical features and Hearing Handicap Inventory for Adults scores.

Participant number	Age	Age of onset	Sex	Symptomsassociated with HL	Type of HL	HHIA score (%)
P1	93	92	M	Tinnitus, pain,vertigo	Bilateral moderate to severe SSNHL	84 (severe handicap)
P2	64	64	M	Tinnitus	Bilateral mildto moderate mixed HL	50 (mild handicap)

HHIA, hearing handicap inventory for adults; HL, hearing loss; M, male; SSNHL, sudden sensorineural hearing loss.

The QoL questionnaire (HHIA) with a score of 84% (P1) indicated that the primary participant’s interactions within their physical, social, emotional and situational subscales were negatively impacted because of the present diagnosis of SOHL. The greater the HHIA score, the more severe the impact on the QoL for those diagnosed with SOHL. Both the primary participants reported that one ear was more affected than the other, P1 (left ear) and P2 (right ear).

Results obtained from the semi-structured interviews (Table 3) were from all the participants, primary and secondary (*N* = 5). Five themes and quotes that describe the impact on the QoL post-SOHL diagnosis are presented in Table 3. The participants reported that their ability to communicate with other individuals was more impacted and negatively influenced how they interacted with their environment. Participants also reported that the SOHL affected their mental well-being and they felt that counselling provided by the audiologists post-SOHL diagnosis was inconsistent and ineffective.

**TABLE 3:** Themes from participants’ interviews.

Objective	Themes	Extracts
Explore the lived experiences of adults diagnosed with SOHL	Prime components of life impacted: Communication with peers at work	‘It’s changed my life drastically because … Especially in business you know I can’t hear what people are saying. Well, I would say my daily work life you know is what is worse affected.’ (P1, male, 93 years old) ‘I would imagine what he’s doing, uh, as a professional. Yeah. Um, is mostly affected.’ (S1, male, 52 years old) ‘Yes. Yes. Because you know, we sit in a meeting, and we talk, and he will say something and he. He cannot hear.’ (S2, female, 53 years old) ‘So sometimes I can hear clear. Sometimes the people they must just talk hard with me.’ (P2, male, 64 years old)
Investigate the impact of SOHL on the quality of life (QoL)	Influences on the nature ofrelationships	‘Let me, you can ask my wife, my wife, it’s with me now when my wife talk to me and she, and I never respond to her back.’ (P2, male, 64 years old) ‘Well, I’m a very soft spoken person. Uh, I often have to speak loud. So that he can hear me.’ (S3, female, 61 years old) ‘And right now he said to me, he’s battling to communicate people can’t communicate with him because he can’t hear what they are saying.’… ‘He’s a very depressed old man and it’s because he’s lonely because he can’t interact with people.’ (S2, female, 53 years old)
Establish facilitators of emotional and social aspects of counselling provided by audiologists post-SOHL diagnosis	Mental wellness and emotional stability: repressed personality and irritability	‘Yes definitely. Definitely because he’s, he’s a lot more subdued. Um, you know, yes. So definitely there has been, it has impacted him quite greatly.’ (S1, male, 52 years old)
	Influences on social behaviours	‘It’s also very bad because you know when I’m with other people I can’t hear what they are saying and I’m tired of saying “yes yes” when I don’t know what is going on around me.’ (P1, male, 93 years old) ‘… he’s not really enjoying it because he’s, he doesn’t know what’s going on. Yeah.’ (S2, female, 53 years old)
Effective and ineffective counselling: effective informational counselling and ineffective patient and family-centred counselling	After you complete the appointment at the University of the Witwatersrand did you feel like you were counselled properly or at all by your audiologist? So, did they sit you down and explain to you properly that this is your hearing loss, this could be the possible causes and this is how we can manage it?	‘Yes, yes.’ (P1, male, 93 years old) ‘Ok so they explain to me my results, my sister, then they tell me I have to go to ENT. When I went to hospital, they said they having an issue and they’ll contact me to give me a booking. I’m still waiting for that my sister.’ (P2, male, 64 years old)
	Did they counsel you in terms of your personal emotions and how you felt about your hearing loss? So, did they ask you whilst they were giving you this information how are you feeling about all of this Mr X etc.?	‘No, no not really.’ (P1, male, 93 years old) ‘Nothing. They didn’t.’ (P2, male, 64 years old)

SOHL, Sudden onset hearing loss.

## Discussion

As diagnostic audiometry does not offer a holistic picture of the impact of HI on the individual’s daily function, the hearing handicap tools that went beyond diagnostic audiometry were designed to integrate interventions and treatment aspects that offered person-centred care (PCC) and family-centred care (FCC). Thus, the study findings aided in the understanding of the impact on the QoL and the tools thereof which were specific for adults diagnosed with SOHL. Although our study sample was small, this was relative to the widely reported incidence rates of 5–20 individuals/100 000 persons/year (Elmoursy et al., 2023). The audiogram findings revealed different hearing sensitivity levels for the primary participants and their perceived hearing handicap may have been influenced by their age and age of SOHL diagnosis which impacted their QoL.

### Impact on communication

The individual’s age and age of SOHL diagnosis as indicated by the HHIA scores estimate the hearing handicap and impact on communication. Our study’s primary participants obtained 84% (P1) and 50% (P2), respectively, and reported that they struggled mostly with communicating with family members at home and peers at work, who formed part of their microsystem. A study by Härkönen et al. (2017) agrees with our findings that high HHIA scores may be linked to secondary factors such as tinnitus, vertigo and otalgia, which were reported by our primary participants. While a study by Kuhn et al. (2011) reported that treatment and prognosis for hearing recovery were dependent on one’s age, severity of SOHL, vertigo and the audiogram configuration, these factors seemed to increase the primary participants’ perception of their hearing handicap. These risks were also indicated in our findings (Table 2) and were in agreement with the findings previously reported by Kuhn et al. (2011). Although the age of P1 (93) may be associated with age-related hearing loss, the perceived severity of the HI was associated with tinnitus, pain, vertigo and the SOHL diagnosis. Recent studies have attributed SOHL to medical conditions such as HIV and coronavirus disease 2019 (COVID-19) (Elmoursy et al., 2023; Frosolini et al., 2022) but these were not reported by our study participants. Thus, successful informational counselling and aural rehabilitation intended to improve the communication abilities of adults diagnosed with SOHL should be informed by patient-specific audiological data on the age of onset and associated risk factors such as tinnitus, vertigo, medical conditions and type of hearing loss. Furthermore, future studies should consider probing patients about their history of COVID-19 infections as part of the case history interview.

### Mental and emotional well-being

Individuals diagnosed with SOHL experience life differently following the SOHL diagnosis; however, factors such as anxiety and depression were associated with hearing loss and affected the individuals’ QoL (Arslan et al., 2018). In our study, although P1 aged 93 years may seem old, he was still actively working, and thus, the SOHL diagnosis may have possibly triggered job insecurity and subsequent experiences of anxiety and depression. In addition, the secondary participants further emphasised the primary participants’ impact on their well-being that following SOHL diagnosis, the primary participants were withdrawn and avoided various communication spaces, which reduced their social interactions and affected their relationships with those within their microsystems. Our findings were similar to those reported by Arslan et al. (2018), who confirmed their participants’ anxiety and depression high scores calculated from the Beck Anxiety Scale (BAS) and the Beck Depression Inventory (BDI) that were conducted following their participants’ initial audiology visit. Therefore, participants’ information about their mental health and well-being deducted from interviews and scores obtained from the hearing handicap scales may be useful intervention measurements when integrated into counselling and used to guide the appropriate referrals.

The HHIA as a quantitative measure should be augmented with a qualitative interview schedule that is context specific for a successful intervention of SOHL. The score of 84% obtained from P1’s HHIA indicated a severe impact on the participant’s QoL. This score is linked with a significant negative impact on one’s QoL, coupled with his age, age of onset of SOHL and reported symptoms of tinnitus, otalgia and vertigo, and the perceived hearing handicap may be severe. The impact on the participants’ QoL affected their communication abilities. Thus, audiologists’ role pre- and post-SOHL diagnosis should integrate patient-and-family-centred counselling as support for those diagnosed with SOHL.

The responses obtained from the individuals who represent the microsystem of the individual diagnosed with SOHL are crucial and inform the external perspective on the QoL and impact the interrelationships of those diagnosed with SOHL. Data obtained from the secondary participants consequently provided in-depth insight into the primary participants’ communication struggles that were not so much reported by the primary participants themselves. Insight knowledge was specifically with regard to the impact of hearing loss on social behaviour, mental wellness and emotional instability, which were more visible to secondary participants. Thus, involving a holistic microsystem type of aural rehabilitation should be reinforced to improve patient-specific hearing healthcare support.

### Impact on counselling

Literature has clearly defined the difference between informational counselling and patient-and-family needs counselling (Meibos, 2018). Meibos (2018) further reported that audiologists were more inclined to focus on informational counselling and paid less attention to the patient-and-family needs (Meibos, 2018). The participants in our study reported that audiologists provided inadequate informational counselling following the SOHL diagnosis, and there was a lack of guidance and follow-up support. These were the clinical competency gaps for audiologists that were also reported by Meibos (2018) which were essential for PCC and FCC practices (ASHA, 2020). Thus, the lack of patient-and-family-centred counselling was a barrier that impacted the participants’ QoL following SOHL diagnosis. Thus, effective interventions offered by audiologists should have PCC and FCC aspects of counselling.

## Conclusion

The integrated interpretation of the study findings drawn from the convergent parallel methods revealed that the lived experiences of adults diagnosed with SOHL and the impact on their QoL varied from person to person and were influenced by one’s age, age of SOHL diagnosis, audiological symptoms such as tinnitus, vertigo and otalgia, and the type of hearing loss. In addition, there was no specific etiological cause for SOHL in our findings. Our study findings demonstrated a link between the HHIA scores and the themes deducted from the interviews. Both elements indicated that the impact on the QoL post-SOHL diagnosis severely affected the participants’ communication abilities with individuals in their microsystem, as well as added attributes that affected their mental well-being. The findings obtained may be site-specific, but the data collection methods and tools used in our study may be generally used by other sites to improve practice management and hearing healthcare for adults at risk for and those diagnosed with rare audiological conditions such as SOHL. Finally, the convergent parallel methods of data collection and findings in our study may be used as a pilot for future studies to consider using for other study populations and to validate our study findings.

### Limitations and strengths of the study

It has been established through a review of literature that SOHL is rare in nature when compared to other types of hearing impairment, hence our small study sample; this was noted as a trend reported in previous studies yet may be seen as a limitation. Nevertheless, the convergent parallel methods of data collection used in our study favour scientific rigour, especially when studying a known rare medical condition (Demir & Pismek, 2018). Although transferability and generalisability of the study findings may not be possible (Smith, 2018), lessons from our methodology may have potential relevance and value in the case management of other rare audiology cases. We accessed our data from the university audiology clinic only; however, we acknowledge that this type of clinic site was not necessarily contextually representative, and hence, our findings may not be generalised. Using the HHIA tool and qualitative interviews allowed for triangulation during data analysis, and this was our study strength that showed scientific rigour.

### Recommendations for practice

Counselling aids an individual not only to accept and understand their hearing loss but also to navigate their daily functions. Daily functioning activities encompass functioning in physical spaces and are inclusive of one’s social behaviours, mental wellness and emotional stability and maintaining functional relationships. Therefore, informational counselling that integrated PCC and FCC may benefit those diagnosed with SOHL and individuals in their microsystems.

The intentional and combined use of the HHIA and qualitative interviews by audiologists during aural rehabilitation post-SOHL diagnosis could be facilitators used to improve the efficiency of patient-centred interventions and add value to their QoL instruments.

## Appendix 1

### Interview guide

#### The experiences of individuals with sudden onset hearing loss: Exploring QoL post-diagnosis

**Research interview questions:**

This interview is semi-structured, and hence, these questions encompass the main idea of the information I want to obtain out of the interviews. Depending on each participant, I may ask additional questions and probe respectively. These questions, however, are questions that will be asked to every participant. The questions are mostly open-ended so as to gain maximum personal insight from each participant:

1. For how many years have you been diagnosed with your sudden hearing loss?
2. At what age you were diagnosed with your hearing loss?
3. In your experience, how did your sudden hearing loss change the way you used to live your life?
4. Is there something specific in your life (like a particular activity or relationship) that you feel had been most affected by your hearing loss as compared to everything else?
5. Personally, how would you describe the impact your hearing loss had on your social life?
6. How would you say the hearing loss impacted your mental health and well-being?
7. Do you feel as if you were counselled appropriately or at all by your audiologist throughout your hearing loss journey?
8. Do you use hearing aids or any assistive devices?
9. Would you say that with the assistive device, your daily life and social life are the same as they were before your hearing loss?

Depending on the client’s responses, probing questions will differ.

## References

[CIT0001] Alexander, T.H., & Harris, J.P. (2013). Incidence of sudden sensorineural hearing loss. *Otology & Neurotology*, 34(9), 1586–1589. 10.1097/mao.0000000000000222 24232060

[CIT0002] American Speech-Language-Hearing Association (ASHA). (2020). *Person-Centered Care in Audiology*. Retrieved September 03 2023, from https://www.asha.org/aud/Person-Centered-Care-in-Audiology/

[CIT0003] Arslan, F., Aydemir, E., Kaya, Y.S., Arslan, H., & Durmaz, A. (2018). Anxiety and depression in patients with sudden one-sided hearing loss. *Ear, Nose, & Throat Journal*, 97(10–11), E7–E10. 10.1177/0145561318097010-1101 30481848

[CIT0004] Bakas, T., McLennon, S.M., Carpenter, J.S., Buelow, J.M., Otte, J.L., Hanna, K.M., Ellett, M.L., Hadler, K.A., & Welch, J.L. (2012). Systematic review of health-related quality of life models. *Health and Quality of Life Outcomes*, 10(1), 134. 10.1186/1477-7525-10-134 23158687 PMC3548743

[CIT0005] Braun, V., & Clarke, V. (2012). Thematic analysis. *APA Handbook of Research Methods in Psychology, Vol 2: Research Designs: Quantitative, Qualitative, Neuropsychological, and Biological*, 2, 57–71. 10.1037/13620-004

[CIT0006] Chau, J.K., Lin, J.R.J., Atashband, S., Irvine, R.A., Westerberg, B.D. (2010). Systematic review of the evidence for the etiology of adult sudden sensorineural hearing loss. *Laryngoscope*, 120(5), 1011–1021. 10.1002/lary.20873 20422698

[CIT0007] Creswell, J.W., & Plano Clark, V.L. (2011). *Designing and conducting mixed methods research* (2nd ed). Sage.

[CIT0008] Crowe, S., Cresswell, K., Robertson, A., Huby, G., Avery, A., & Sheikh, A. (2011). The case study approach. *BMC Medical Research Methodology*, 11(1), 100. 10.1186/1471-2288-11-100 21707982 PMC3141799

[CIT0009] Dalton, D.S., Cruickshanks, K.J., Klein, B.E.K., Klein, R., Wiley, T.L., & Nondahl, D.M. (2003). The impact of hearing loss on quality of life in older adults. *The Gerontologist*, 43(5), 661–668. 10.1093/geront/43.5.661 14570962

[CIT0010] Demir, S.B., & Pismek, N. (2018). A convergent parallel mixed-methods study of controversial issues in social studies classes: A clash of ideologies. *Educational Sciences: Theory & Practice*, 18, 119–149.

[CIT0011] Elmoursy, M.M., Bakr, M.S., Mohamed, E.S., & Ragaee, M.A. (2023). The incidence of sudden sensorineural hearing loss (SSNHL) in COVID-19 patients in tertiary care referral units. *SN Comprehensive Clinical Medicine*, 5(1), 87. 10.1007/s42399-023-01420-4 36845674 PMC9942031

[CIT0012] Foden, N., Mehta, N., & Joseph, T. (2013). Sudden onset hearing loss causes, investigations and management. *Australian Family Physician*, 29(9). Retrieved from https://www.racgp.org.au/afp/2013/september/sudden-onset-hearing-loss 24024225

[CIT0013] Frosolini, A., Franz, L., Daloiso, A., De Filippis, C., & Marioni, G. (2022). Sudden sensorineural hearing loss in the COVID-19 pandemic: A systematic review and meta-analysis. *Diagnostics (Basel, Switzerland)*, 12(12), 3139. 10.3390/diagnostics12123139 36553146 PMC9777296

[CIT0014] Gondim, L.M.A., Balen, S.A., Zimmermann, K.J., Pagnossin, D.F., Fialho I.M., & Roggia, S.M. (2012). Study of the prevalence of impaired hearing and its determinants in the city of Itajaí, Santa Catarina State, Brazil. *Brazilian Journal of Otorhinolaryngology*, 78(2), 27–34. 10.1590/S1808-86942012000200006 PMC9443891 22499367

[CIT0015] Granberg, S., Pronk, M., Swanepoel, D.W., Kramer, S.E., Hagsten, H., Hjaldahl, J., Möller, C., & Danermark, B. (2014). The ICF core sets for hearing loss project: Functioning and disability from the patient perspective. *International Journal of Audiology*, 53(11), 777–786. 10.3109/14992027.2014.938370 25311099

[CIT0016] Härkönen, K., Kivekäs, I., Rautiainen, M., Kotti, V., & Vasama, J.P. (2017). Quality of life and hearing eight years after sudden sensorineural hearing loss. *The Laryngoscope*, 127(4), 927–931. 10.1002/lary.26133 27328455

[CIT0017] Joubert, K., & Botha, D. (2019). Contributing factors to high prevalence of hearing impairment in the Elias Motsoaledi Local Municipal area, South Africa: A rural perspective. *South African Journal of Communication Disorders*, 66(1), e1–e7. 10.4102/sajcd.v66i1.611 PMC6407449 30843412

[CIT0018] Korstjens, I., & Moser, A. (2017). Series: Practical guidance to qualitative research. Part 4: Trustworthiness and publishing. *European Journal of General Practice*, 24(1), 120–124. 10.1080/13814788.2017.1375092 29202616 PMC8816392

[CIT0019] Kuhn, M., Heman-Ackah, S.E., Shaikh, J.A., & Roehm, P.C. (2011). Sudden sensorineural hearing loss: A review of diagnosis, treatment, and prognosis. *Trends in Amplification*, 15(3), 91–105. 10.1177/1084713811408349 21606048 PMC4040829

[CIT0020] Lin, F.R., & Albert, M. (2014). Hearing loss and dementia – Who is listening? *Aging & Mental Health*, 18(6), 671–673. 10.1080/13607863.2014.915924 24875093 PMC4075051

[CIT0021] Meibos, A. (2018). *Counselling competencies in audiology: Important knowledge, skills and attitudes*, All graduate theses and dissertations. Utah State University.

[CIT0022] National Month of Deaf People. (2020). *Western Cape Government*. Retrieved from https://www.westerncape.gov.za/general-publication/national-month-deaf-people

[CIT0023] Newman, C.W., Weinstein, B.E., Jacobson, G.P., & Hug, G.A. (1990). The hearing handicap inventory for adults: Psychometric adequacy and audiometric correlates. *Ear and Hearing*, 11(6), 430–433. 10.1097/00003446-199012000-00004 2073976

[CIT0024] Pearson, N., Muñoz, K., Landon, T.J., & Corbin-Lewis, K. (2019). Counseling skills in audiology. *The Hearing Journal*, 72(3), 50. 10.1097/01.hj.0000554354.53822.78

[CIT0025] Scarinci, N., Worrall, L., & Hickson, L. (2009). The ICF and third-party disability: Its application to spouses of older people with hearing impairment. *Disability and Rehabilitation*, 25(31), 2088–2100. 10.3109/09638280902927028 19888839

[CIT0026] Schreiber, B.E., Agrup, C., Haskard, D.O., & Luxon, L.M. (2010). Sudden sensorineural hearing loss. *Lancet (London, England)*, 375(9721), 1203–1211. 10.1016/S0140-6736(09)62071-7 20362815

[CIT0027] Smith, B. (2018). Generalizability in qualitative research: Misunderstandings, opportunities and recommendations for the sport and exercise sciences. *Qualitative Research in Sport, Exercise and Health*, 10(1), 137–149. 10.1080/2159676X.2017.1393221

[CIT0028] Ventry, I.M., & Weinstein, B.E. (1983). Identification of elderly people with hearing problems. *ASHA*, 25, 37–42. 6626295

[CIT0029] World Health Organization. (2021). *World report on hearing*. WHO. Retrieved from http://apps.who.int/iris

[CIT0030] World Health Organization Geneva. (2013). *How to use the ICF – A practical manual for using the International Classification of Functioning, Disability and Health (ICF) Exposure draft for comment*. World Health Organization. Retrieved from https://www.who.int/classifications/drafticfpracticalmanual.pdf

